# Sweet/Fat Preference Taste in Subjects Who are Lean, Obese and Very Obese

**DOI:** 10.1007/s11095-020-02968-9

**Published:** 2020-11-19

**Authors:** Jennifer Leohr, Maria C. Kjellsson

**Affiliations:** 1 Department of Pharmacokinetics/Pharmacodynamics, Lilly Research Laboratories, Lilly Corporate Center, Indianapolis, Indiana 46285 USA; 2 Department of Pharmacy, Pharmacometrics Research Group, Department of Pharmaceutical Biosciences, Uppsala University, Box 580, SE-751 23 Uppsala, Sweden

**Keywords:** categorical modeling, fat, hedonic, obesity, preference, sugar

## Abstract

**Purpose:**

This study assessed the perception of sweetness, creaminess, and pleasantness from a sweet/fat preference test in subjects who are lean (BMI: 19–25), obese (BMI: 30–33) or very obese (BMI: 34–40) using categorical modeling.

**Methods:**

Subjects tasted 16 dairy solutions consisting of 0%, 3.5%, 11.3% and 37.5% fat and each containing 0%, 5%, 10%, or 20% sugar and rated them for sweetness, creaminess and pleasantness.

**Results:**

A proportional odds model described the perception of sweetness using an Emax for the effect of sugar and a linear effect for fat. Perception of creaminess was dependent on the fat and sugar content and was described with proportional odds model with linear effects of sugar and fat. Perception of pleasantness increased with sugar and fat but decreased in solutions containing 37.5% fat. A differential odds model using an Emax model for fat and sugar with a negative interaction between them allowed the sugar content to be less than proportional and the fat content to be greater than proportional for pleasantness.

**Conclusions:**

Application of modeling provided understanding of these complex interactions of sugar and fat on the perception of sweetness, creaminess, and pleasantness and provides a tool to investigate obesity and pharmacological intervention.

**Supplementary Information:**

The online version contains supplementary material available at 10.1007/s11095-020-02968-9.

## Introduction

Obesity is recognized as a major health epidemic worldwide (1) and is a disorder of chronic positive energy balance, whereby excess of energy intake beyond energy utilization leads to an increase in adipose tissue.

Chronic over-eating leading to the development of obesity can be categorized as an addictive behavior, similar to other substance disorders in which craving, and reward plays a key role (2). This is supported by a meta-analysis of functional MRI studies which has shown that food and drugs activate similar brain regions (3). Additionally, similar brain abnormalities during the presentation of stimuli in reward and salience progressing has been observed between obesity and substance addiction (4).

The motivation to eat is closely related to the hedonic reward of food which is linked to the sensory perception of food in the mouth (5). It is believed that obesity is due to the overconsumption of highly processed food with “empty calories”, i.e. high sugar and fat content and few nutrients. Correspondingly, it has been found that overweight participants crave high-caloric, high palatable foods more frequently at non-eating moments than normal-weight participants (6).

Studies assessing the hedonic response using a sugar/fat preference test consisting of diary solutions with varying levels of fat and sugar have been conducted in several different populations (7–9). However, the results are inconsistent. Salbe *et al.* showed that in Pima Indians, the degree of hedonic response elicited by preferred solutions was positively correlated with weight gain (7). Yet, Pima Indians had a lower hedonic response to sweet and creamy solutions compare to white participants in the study (7). Additionally, the hedonic response was not associated with body size or adiposity (7). This is a somewhat surprising given that the liking of foods containing high amounts of sugar and fat is considered the driver for weight gain and obesity. In contrast, another study found that the hedonic response which corresponded to the preferred sugar to fat ratio was negatively correlated with body mass index (BMI) (8). Likewise, Drewnowski *et al.* showed that subjects who are binge eaters had a higher preference rating for solutions containing 16 and 32% sugar than control subjects (9). While the preference rating for fat level was higher in people who are binge eaters compared to controls, it was found to not be significant (9).

The sugar/fat preference test for hedonic response has also been used to assess pharmacologic intervention using opioid peptides, which regulate food intake (9). Naloxone, an opioid antagonist, reduces food intake in both normal-weight (10) and obese subjects (11), as well as reduced the meal size in women who are suffering from bulimia (12). In contrast, butorphanol, an opioid agonist, increases food intake in humans (13). It is thought that the primary role of the opioid system is in regulation of food intake of highly palatable (14) or high-fat food (15). Correspondingly, naloxone infusions, compared to saline, significantly reduced hedonic preferences for both sugar and fat in both subjects who are binge eaters and control subjects (9). Butorphanol marginally reduced hedonic preference in subjects who are binge eaters, while it increased the hedonic preference in control subjects (9). These findings are encouraging as the application of this test within the field of obesity could better define individuals who are more likely to respond to specific pharmacological treatment and provide a way to tailor drug therapies.

Many of these studies used an ANOVA to analyze the categorical outcome measures. ANOVA have been showed to be inappropriate for categorical outcome measures and can yield spurious results (16). A better approach is the use of logistic model, which provides a variety of advantages over regression analysis, such as more information on the direction and size of effect (16). Mixed-effects logistic models provides an additional benefit over the logistic models, as random effects are used to acknowledge inter-individual variability (IIV) with longitudinal categorical outcome measures. Analyzing categorical data using nonlinear mixed-effects logistic modeling was introduced in the field of pharmacokinetic-pharmacodynamic (PKPD) modeling by Lewis Sheiner in 1994 (17). It has since been used in a wide variety of therapeutic areas for both clinical efficacy and side effects, such as pain relief (18), nicotine craving scores (19), grade of neutropenia (20) and sedation scales (21). Modeling allows the incorporation of the level of fat and sugar on the perception of sweetness, creaminess and hedonic scoring of the tests as well as simultaneous assessment of interaction of sugar and fat on the perception of sweetness, creaminess and preference (hedonic) scoring. Some studies looked at only the level of sugar on sweetness or the level of fat on creaminess and neglected the possibility of both contributing to the sweetness or creaminess score (9). This modeling approach also allow the assessment of various covariates, such as body weight, which could also influence the scoring. Lastly, this approach also provided the ability to use the model to run simulations to inform future clinical development.

The aim of this study was to assess perception of sweetness, creaminess, and pleasantness using data from a sweet/fat preference test in subjects who are lean to subjects who are obese or very obese using categorical modeling.

## Subjects and Methods

### Participants

Sixty-four healthy subjects, 31 males and 33 females, participated in the study and were categorized into three populations: lean (BMI of 19–25), obese (BMI of 30–33), and very obese (BMI of 34–40). The enrollment aimed to have approximately 20 subjects per BMI category. All were healthy subjects age 26–45 years old with no known cardio-metabolic disease, drug dependency, taking essential medication or supplements, or weight loss >5 kg in last 6 months. Demographics of the subjects by population is presented in Table I.

**Table I:** Summary of Subject Characteristics by Population

Study population		BMI (kg/m^2^)	Fat (%)	WT (kg)	HC (cm)	WC (cm)	FPG (mg/dL)	HOMA-IR
Lean	Mean	22.7	10.6	70.4	96.2	80.0	95.5	0.41
*N* = 24 (7F, 17 M)	95% CI	22.1;23.4	7.83;13.3	67.0;73.7	93.7;98.8	77.6;82.3	92.1;99.0	0.27;0.55
Obese	Mean	31.8	32.0	89.0	112	98.9	101	0.75
*N* = 23 (16F, 7 M)	95% CI	31.3;32.3	29.2;34.8	85.3;85.3	110;114	96.0;102	97.2;105	0.60;0.89
Very obese	Mean	36.6	40.6	107	120	116	104	1.63
*N* = 17 (10F, 7 M)	95% CI	35.7;37.4	37.4;43.7	101;113	118;123	112;119	99.5;108	1.23;2.03

*BMI* body mass index, *Fat* body fat, *F* female, *WT* weight, *HC* hip circumference, *WC* waist circumference, *FPG* fasting plasma glucose (mg/dl), *HOMA-IR* Homeostasis model assessment for Insulin Resistance, HOMA-IR, *M* male

### Study Design

This was a single center study and approved by the Indiana University Institutional Review Board in 2006. All patients gave written informed consent. All procedures performed in studies involving human participants were in accordance with the ethical standards of the institutional and/or national research committee and with the 1964 Helsinki declaration and its later amendments or comparable ethical standards. Public registration of the trial was not required at the time of the trial.

### Intervention

Subjects were admitted to the Clinical Research Unit (CRU), and their body weight, and height measured. The BMI was calculated using an average of three body weights measurements (in kg) divided by the height collected at admission (in m) squared. Additionally, the subjects’ body composition were assessed, using quantitative nuclear magnetic resonance (qNMR; EchoMRI-AH™ qNMR device, Houston, USA). Two qNMR scans were done consecutively with removal of the subject from the device for repositioning between each scan. The scan duration of each qNMR measurement was ≤5 min. For both body weight and qNMR measurement, subjects were dressed only in light clothing and asked to attempt to empty their bowel and bladder prior to the measurement.

A sugar/fat preference test (SFPT) was administered consisting of 16 solutions containing: skim milk (0% fat), whole milk (3.5% fat), half and half (11.3% fat) or cream (37.5% fat) each containing 0%, 5%, 10%, or 20% sugar by weight (Table II). Consistent with other publications using a SFPT (8,9,22,23), the solutions were kept cold and presented in a dark room in a solid cup to prevent visual inspection of the solutions. Subjects sipped, tasted each solution, and expectorate it without swallowing. Each solution was rated immediately for sweetness, creaminess, and pleasantness on separate scales anchored with descriptors of “not at all” (score of 1) and “extremely” (score of 9). A water rinse occurred between each solution’s evaluation.

**Table II:** Summary of Experimental Solutions in Sugar/Fat Preference Test

	Fat Content in Test Solutions				
		0%	3.5% (whole milk)	11.3% (half & half)	37.5% (cream)
Sugar Content in Test Solutions	0%	F1S1	F2S1	F3S1	F4S1
	5%	F1S2	F2S2	F3S2	F4S2
	10%	F1S3	F2S3	F3S3	F4S3
	20%	F1S4	F2S4	F3S4	F4S4

Legend: *F* =fat, *S* =Sugar

**Fig. 1:**
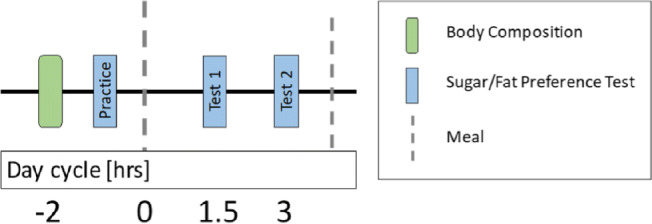
Study procedure diagram.

Prior to lunch, a practice test was conducted where all of solutions were provided to from lowest to highest concentration of fat/sugar to the subject to orientate them to entire range of sugar and fat in the solutions prior to the testing periods (Fig. 1). The data for this period was recorded, but not used in the analysis. A standardize lunch meal was provided to avoid any influence of hunger on scoring of the solutions. Approximately 90 min following the lunch meal, the 16 solutions were presented in random order and scored, as described above. A second assessment period occurred within 90 min of the first test and the solution were presented in random order which differed from the prior session.

## Statistical Analysis

### Variability and Reproducibility

To assess the reproducibility between Test 1 and Test 2, the correlation for each of the solution across the three scores (sweetness, creaminess, and pleasantness) was calculated using Spearman’s rank test.

### Graphical Analysis of Response by Population

The mean and standard error were calculated for the 16 solutions for each population (lean, obese, and very obese) across the different scores (sweetness, creaminess, and pleasantness). The responses for each score was compared graphically between the three populations.

### Mixed-Effects Logistic Modeling

In modeling of categorical outcome, the probability of an observation, rather than the numerical value of the outcome itself, is used in the analysis. Thus, in a logistic model, the cumulative distribution of the probability of the outcomes is assumed to be a logistic distribution.

To characterize the sweetness, creaminess, and pleasantness score, proportional and differential odds models were investigated. The proportional odds model assumes that the effect size of a predictor (e.g. level of sugar or fat) is the same for all categories, meaning that an increase/decrease due to a change in a predictor will affect the log odds of all categories equally (18).

In the differential odds model, this assumption is relaxed allowing categories to be affected unequally by changes in a predictor (24). Importantly, the differential odds model can collapse into the proportional odds model, if differential odds are not supported by the data. This flexibility has shown to be valuable in analyzing sedation data (24–26) and ocular itching scores (27).

Mixed-effect modeling was used to analyze the scores of the sugar/fat preference tests, using NONMEM (version VII) (ICON Development Solutions) (28) and Perl-speaks-NONMEM (PsN) (29) as the modeling environment. Laplacian estimation method with the likelihood option was used. Model discrimination was guided by visual predictive checks (VPCs), likelihood ratio test based on objective function value (OFV; *p* value = 0.01), and precision of parameter estimates. Additionally, a parsimonious approach was used where the simpler (i.e. fewer parameters), of two competing models with the same explanatory predictive power, was selected.

The predictive properties of the model were assessed by performing VPCs using PsN and Xpose in R (30), simulating 1000 study replicates with the same design characteristics as those of the original study. Detail of the categorical modeling calculations are presented in the supplemental.

## Results

### Variability in Sugar/Fat Preference Test

**Fig. 2:**
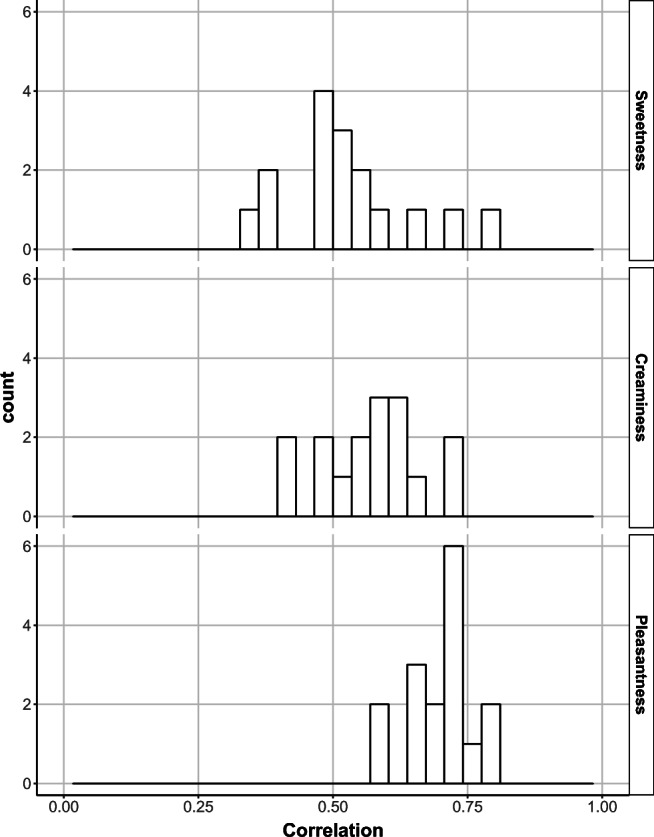
Distributions of correlations between the two test periods from the 16 solutions for Sweetness (top panel), Creaminess (middle panel) and Pleasantness (bottom panel).

The reproducibility of the scoring for each solution for sweetness, creaminess and pleasantness is presented in Fig. 2. A perfect correlation of 1 would indicate that the same score (1–9) was provided on both test periods for every subject for each solution. Among the 3 scores, the pleasantness scores were the most reproducible with an average correlation across all solutions of 0.70 (range 0.59–0.79), compared with sweetness and creaminess which had a correlation of 0.53 (range 0.35–0.78) and 0.57 (range 0.42–0.72), respectively (Fig. 2, Table S1). The sweetness score correlation was highest at low sugar content and decreased with increasing sugar concentration and a similar trend was observed for creaminess scores correlations with fat concentrations. In contrast, the correlation increases for sweetness with increasing fat (results not shown).

### Comparison of Score of Sweetness, Creaminess, and Pleasantness by Population

**Fig. 3:**
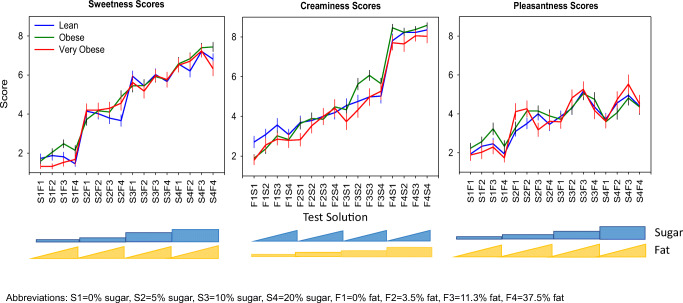
Mean ± standard error of mean of score for sweetness (left), creaminess (middle), and pleasantness (right) by population.

Ratings of sweetness, creaminess, and pleasantness were assessed across the three populations (subjects who are lean, obese, and very obese). The mean score for each solution by population is presented in Fig. 3. Overall, the scoring between the populations were similar.

All populations were able to distinguish the different levels of sugar in the solutions. (Fig. 3, left panel). The average sweetness score increased incrementally with the solutions containing 0% to 10% sugar (mean of 2, 4 and 6 for 0, 5% and 10% sugar, respectively), except between 10% and 20% sugar where the sweetness score only increased slightly (mean of 7.5 and 8, respectively). Solutions containing less than 20% sugar were not influenced by the level of fat in the solution. However, the sweetness score increased with the fat content in the solutions containing 20% sugar, except with the highest level of fat (37.5%). This lack of increase in sweetness scoring was not due to subjects scoring the maximum of 9 (Fig. S1).

In contrast, the creaminess score gradually increased with solutions containing up to 11% fat and the influence of both the fat and sugar levels increased the perception of creaminess (Fig. 3, middle panel). Only the highest level of fat (37.5%) did not show an effect of sugar on the creaminess score. This was due to the majority of subjects providing the maximum score of 9 for the highest fat level without any sugar; therefore, limiting the ability to increase the score with the addition of the level of sugar (Fig. S2). Generally, subjects who are very obese tended to score the solutions lower than those subjects who are lean or obese.

The pleasantness scoring was more complex than both the sweetness and creaminess scores. The score for pleasantness increased with increasing levels of sugar and fat, except for solutions containing 37.5% fat, which were scored about the same as the solutions containing less fat; generally, less pleasant. The pleasantness score was similar between solutions containing 10% and 20% sugar (Fig. 3, right panel). Notably, the pleasantness score was not close to the maximum score of 9 and the similarities between the two highest sugar concentrations can thus not be explained by a ceiling effect, as observed with the creaminess score (Figs. S3 and S4).

### Characterization of Score of Sweetness, Creaminess, and Pleasantness by Population Modeling Analysis

**Fig. 4:**
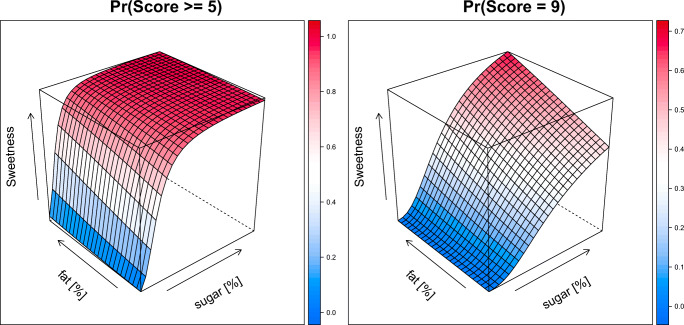
Surface plots of the probability of scoring a 5 or higher (left) and a 9 (right) for sweetness based on the level of sugar [%], and level fat [%] based on population model. Color indicates the probably (scale on the right side of each panel).

The sweetness score was well described with a proportional odds model (see supplemental: Details on modeling and Table S2). The effect of sugar on the sweetness score was best described by an Emax model. The model also identified a linear effect for the fat content on the sweetness score. As illustrated in Fig. 4 (left), the probability of scoring a 5 or higher is mainly driven by the sugar content. There is only a minor effect of the fat content in the solution on the sweetness score. For the probability of the maximum score of 9 (Fig. 4, right), the effect of both sugar and fat content is more apparent.

**Fig. 5:**
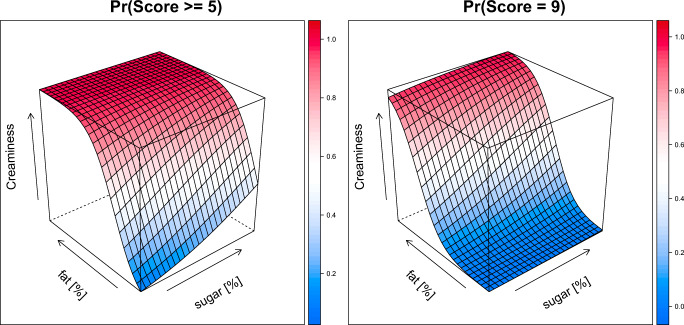
Surface plots of the probability of scoring a 5 or higher (left) and a 9 (right) for creaminess based on the level of sugar [%], and level of fat [%] based on population model. Color indicates the probably (scale on the right side of each panel).

**Fig. 6:**
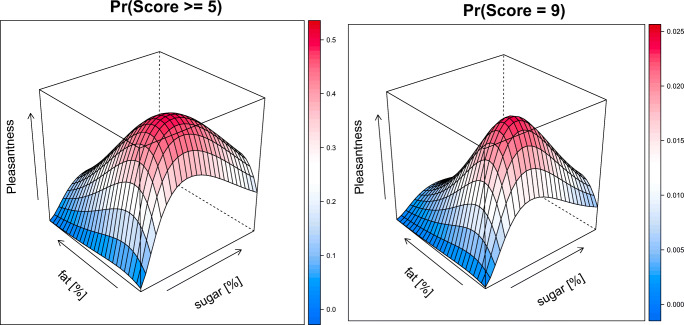
Surface plots of the probability of scoring a 5 or higher (left) and a 9 (right) for pleasantness based on the level of sugar [%], and level of fat [%] based on population model. Color indicates the probably (scale on the right side of each panel).

The creaminess score was well described with a proportional odds model with linear effects for the level of fat and sugar on the score (see supplemental: Details on modeling and Table S2). The model showed that the probability of scoring a 5 or higher is driven by the level of fat and sugar, with the level of fat contributing more than sugar (Fig. 5, left). In contrast, the probability of a maximum of score of 9 is driven primary from the level of fat, while the level of sugar only slightly increases the probability (Fig. 5, right) A differential odds model allowed for sugar to be less than proportional and level of fat to be greater than proportional on the pleasantness score. The effect of the level of sugar and fat on pleasantness was described by Emax models with a negative interaction between sugar and fat (see supplemental: Details on modeling and Table S2). This is illustrated in Fig. 6, which shows the probability of a pleasantness score of 5 or higher (left panel) and a 9 (right panel). The pleasantness score is more dependent on the level of sugar than the level of fat in solution. However, the solutions are less pleasant with higher levels of fat and sugar. As the highest score of 9 is used for hedonic preference, the most preferred solutions showed as the peak in the plot are solutions containing 10% sugar and low level of fat (3–11%). No significant effect between the populations was identified.

### Model Simulations

**Fig. 7:**
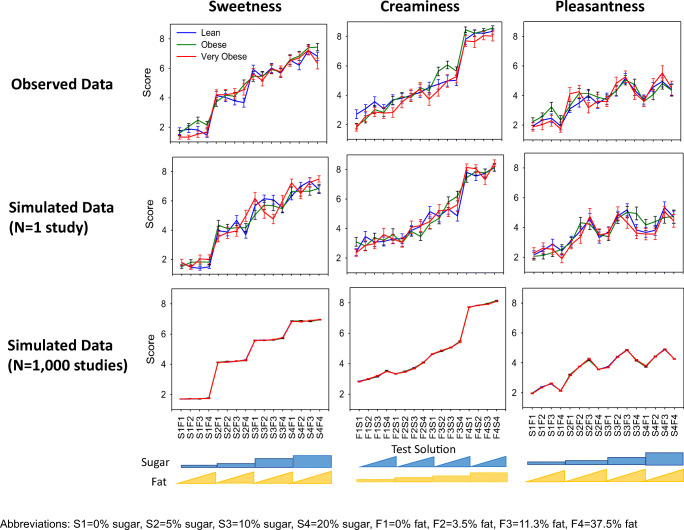
Mean ± standard error of mean of score for sweetness (left), creaminess (middle), and pleasantness (right) by population for the observed data (top), a simulated study (middle) and median of 100 simulated studies (bottom).

To assess the validity of the model to capture the observed data, the final model parameters were used in model to simulate the scoring for the 16 solutions across the 3 populations using the design characteristics of the original study. The simulations showed that the model was able to reflect the observed scoring for the 16 solutions for sweetness, creaminess, and pleasantness for three populations [Fig. 7 top (observed) *vs* middle row (simulated)]. The model was then used with the final parameter estimates to simulate the results of the preference test if conducted 1000 times. Taking the median of these 100 simulated studies highlights the underlying model predictions, without inter-study variability and thus better shows the contribution of sugar and fat within these solutions for the scoring of sweetness, creaminess and pleasantness for these populations (Fig. 7, bottom row).

## Discussion

This was the first study that compared the perception of sweetness, creaminess, and pleasantness using a sweet/fat preference test in subjects who are lean to subjects who are obese or very obese using categorical modeling.

As the 16 solutions within the sugar/fat preference test were presented to the subjects twice for the taste perception rating of sweetness, creaminess and pleasantness, this study also investigated the reproducibility of this test. Among the 3 scores, the pleasantness scores were the most reproducible (mean correlation of 0.70) compare with the sweetness (mean correlation of 0.53) and creaminess scores (mean correlation of 0.57). This aligns well with data reported by Coulon *et al*., showing that pleasantness had better test-retest reliability than the sweetness and creaminess scoring (31). Unlike the data from this study, they reported that sweetness and creaminess scoring were not reproducible (31). There were some differences between the studies. Their study included an additional level of fat (52%) within the preference test, the solutions were rated with a VAS scale of 1–100, instead of 1–9 scale used within this study, and they did not include a practice test. (31). It is likely that difference in the test-retest reliability for the sweetness and creaminess scoring between their study and this study is due to the practice session implemented within this study. This session likely improves the scoring as subjects are orientated to the entire range of sugar and fat in the solutions prior to the testing periods.

The application of categorical modeling to this data provided additional benefits over traditional statistical analysis. Population modeling is more powerful than traditional statistical testing as information is shared across individuals (32,33). The model incorporates the effects of both sugar and fat content on the taste perception rating of sweetness, creaminess and pleasantness, separating the inter-individual variability from inter-test variability, simultaneously assessing additional correlations between the three perception tests. Likewise, the model was able to identify effects which were not apparent by only analyzing the mean data. Additionally, this approach also provided the ability to use the model to run simulations to test other untested combination and to inform future clinical development.

As previously reported (8,22,23,34), the mean creaminess scores moderately increased with fat content in the solutions, then markedly increased with the solutions containing 37.5% fat (cream). Although the increase in the creaminess score was large, it is reflective of the change to a greater level of fat from 11% to 37.5%. The addition of sugar also increased the creaminess score but had less of an effect in the solutions containing 37.5% fat (cream). As the highest level of fat was rated at the maximal score for many of the individuals, the additional effect of sugar on the creaminess score would be reduced due to a ceiling effect. Although the subjects which were very obese tended to perceive the solutions as less creamy than the subjects who are lean or obese, no significant population differences were identified within the modeling for any of the model parameters. Other covariates, such as HOMA, BMI, % body fat, were also not significant. These findings are consistent with others (7,8) who reported that neither adiposity nor BMI was a predictor of creaminess. Additionally, Mela and colleagues found that the mean preferred level of fat across all foods, as an indicator of overall fat preference, was uncorrelated to fat intake (35). Subjects were able to distinguish different levels of sugar in the solutions. The rating of sweetness was mainly influenced by the level of sugar in the solution, as previously reported (8,22,23,34). However, the fat content was found to increase the perception of sweetness within the modeling. This additional effect on the perception of sweetness has not been previously reported. In looking at the data reported from Hynes and colleagues, there is a slight increase in the sweetness score within the response surface plots and the isocontours were not parallel at the highest 35% fat and 20% sugar (34). This suggest some influence of fat on the sweetness score; however, it was not found to be significant based on their analysis (34). Similarly, to the creaminess score, there was no significant effect of population, BMI, % body fat, or HOMA-IR identified within the modeling for any of the model parameters for the perception of sweetness. Like creaminess, these findings are consistent with others who reported that neither adiposity nor BMI was a predictor of sweetness (7,8). Furthermore, a recent study found that the perceived sweetness intensity did not appear to be related to sugar consumption and dietary intake in young adults (36).

There was an increase in mean pleasantness scoring with increasing sugar and fat in all subject groups through solutions containing 11.3% fat (half and half). However, solutions with the highest fat (37.5%) were preferred less than the 11.3% fat solutions. These findings are aligned to what has previously been reported (8). One possible explanation for this is that the highest fat solution may have coated the tongue; thus, blocking the taste of sugar in the solution and resulting in a lower score. This was also observed with the sweetness score where 20% sugar level in the presence of 37.5% fat was scored lower than 11% fat for the subjects who are lean and very obese.

In this study, there were no significant differences in the pleasantness score between subjects who are lean, obese or very obese. As observed in the mean plots, the response across the three populations either overlapped or the response for the lean group was between the response for subjects who were obese and very obese. Additionally, the effect of population, BMI, % body fat or HOMA-IR was not identified as significant within the modeling for any of the model parameters. This differs from another study which found that the degree of hedonic response corresponding to the preferred sugar-to-fat ratio was shown to vary with BMI (8). Their study also included reduced-obese patients who had weighed 99.0 ± 6.9 kg and lost a mean of 31.0 ± 6.7 kg, more than 1 year prior to the start of the study. Our study did not include such a population. Additionally, as seen in their results the uncertainty of preferred sugar-to-fat ratio for subject who are lean was larger than what was observed in subjects who are obese (8). A change in uncertainty could potentially induce a correlation.

In this study, there were no significant differences in the sweetness, creaminess, or pleasantness score between subjects who are lean and those who are obese or very obese. This may be surprising given that obesity is thought to result from an interaction between genetic predisposition and overconsumption of foods high in fat and sugar. However, the perception of pleasantness within the SPFT is measuring “liking”, rather than “wanting”. Liking measures the subject’s preference to consume; however, liking may not correlate to a desire to consume (37). This was illustrated in a study using the sip and spit procedure where discrepancies were found between the sucrose concentration most preferred and the sucrose concentration most consumed (38). Likewise, there has been a lack of associations between sensory measures and dietary intake in diet-taste relationships (35). Additionally, we categorized the groups solely on BMI for this analysis However, BMI and other physical descriptors (i.e. % fat mass, hip circumference or waist circumference) reflect the outcome of obesity rather than the reason. Clearly, there are various causes for obesity including over-eating, lack of physical activity, hormone imbalances, eating disorders (binge-eating), etc. The application of the SPFT and model would be of particular interest in individuals where weight gain is due to a food consumption disorders, such as binge-eating.

### Limitations

The SFPT is performed with liquids which may not be representative of solid food given the additional attributes of texture of fats in solid foods. Thus, extrapolations to solid food may not be valid as there might be other relationships between amount of sugar and fat and the preception of sweetness, creaminess and pleasantness.

The data from this study was generated via a VAS scale and assumes a similar absolute perceived intensity to all individuals. However, as pointed out by Bartoshuk and colleagues, the perceived intensities may vary as they depend on the individual experiences with the substances being judged (38). They suggest that this variation makes across-group comparisons invalid. However, these arguments are valid only for group comparisons, where populations are compared without taking into account an individual’s response. We have applied mixed-effects modeling where an individual’s response is acknowledged through the random effects describing the individual baseline scoring, while sharing the typical response across all individuals. Thus, categorical modeling of VAS scales makes group-comparisons possible, in addition to the higher statistical power as mentioned above.

## Conclusions

The application of categorical modeling to this data provided a powerful approach to further understand these complex interactions of sugar and fat on the perception of sweetness, creaminess, and hedonic response. Although the perception of sweetness, creaminess, and hedonic response was similar between subjects who are lean, obese and very obese, the application of this model could be informative in investigating subcategories of obesity, eating disorders, and pharmacological intervention.

## Supplementary information

ESM 1 (DOCX 167 kb)
