# Supplementary material for: Sweet/Fat Preference Taste in Subjects Who are Lean, Obese and Very Obese
Source: Pharm Res. 2020 Nov 19;37(12):244. doi: 10.1007/s11095-020-02968-9 (PMC7677291; doi:10.1007/s11095-020-02968-9)
Supplement: Supplementary file 1 — (DOCX 167 kb) [file 11095_2020_2968_MOESM1_ESM.docx]

### Supplemental

**Table S1. Correlation between Scores from Two Tests**

| Solution | CreaminesS | Sweetness | Pleasantness |
| --- | --- | --- | --- |
| F1S1 | 0.47 | 0.50 | 0.59 |
| F1S2 | 0.60 | 0.52 | 0.73 |
| F1S3 | 0.53 | 0.35 | 0.71 |
| F1S4 | 0.55 | 0.56 | 0.70 |
| F2S1 | 0.62 | 0.52 | 0.78 |
| F2S2 | 0.72 | 0.53 | 0.79 |
| F2S3 | 0.65 | 0.39 | 0.71 |
| F2S4 | 0.50 | 0.50 | 0.67 |
| F3S1 | 0.54 | 0.74 | 0.71 |
| F3S2 | 0.63 | 0.54 | 0.64 |
| F3S3 | 0.72 | 0.47 | 0.67 |
| F3S4 | 0.62 | 0.38 | 0.60 |
| F4S1 | 0.43 | 0.78 | 0.70 |
| F4S2 | 0.42 | 0.59 | 0.71 |
| F4S3 | 0.59 | 0.47 | 0.71 |
| F4S4 | 0.60 | 0.65 | 0.77 |
| Mean | **0.57** | **0.53** | **0.70** |
| Range | **0.35-0.78** | **0.42-0.72** | **0.59-0.79** |

Legend: F1 = skim (0% fat), F2 = whole milk (3.5% fat), F3 = half and half (11.3% fat), F4 = cream

(37.5% fat), S1 = 0% sugar, S2 = 5% sugar, S3 = 10% sugar, S4 = 20% sugar.

**Details of Modeling**

The section below describes in detail the modeling of the three scores: Sweetness, Creaminess and Pleasantness. The proportional odds model was used to model Sweetness and Creaminess while the differential odds model was used to model Pleasantness.

The scores of the measurement (Sweetness, Creaminess or Pleasantness) all ranged from 1 to 9 and all individuals had 16x2 observations (16 different sugar/fat combinations, administered twice).

***The proportional odds model (Sweetness and Creaminess)***

If *y_i_* = *(*y*_i_*_1_*,* y*_i_*_2_*, . . .,*y*_i32_)* is the vector of categorical response for the *i*^th^ individual with *32* observations, then the probability that an observation, *y_in_* is greater than or equal to the score *j* (where *j= 2 … 9*) has the following general structure:

$Pr\left( y_{in}\geq j | \eta_{i} \right)=\frac{e^{{logit}_{ij}}}{1+e^{{logit}_{ij}}}$ (1)

where

${logit}_{ij}=f_{j}\left( \right)+ \eta_{i};j=2,\ldots,I$ (2)

*η_i_* is the random effect, describing inter-individual differences. All random effects belong to a normal distribution with zero mean and variance  *ω*^2^, which is estimated. The function, *f_j_( )* describes baseline probabilities (on logit scale) and effects of predictors, e.g. covariates, doses and time, as follows:

$$f_{2}\left( \right)=\alpha_{2}+g\left( X \right)$$

$$f_{3}\left( \right)=\alpha_{2}+\alpha_{2\to3}+g\left( X \right)$$

$f_{4}\left( \right)=\alpha_{2}+\alpha_{2\to3}+\alpha_{3\to4}+g\left( X \right)$ (3)

*…*

$$f_{9}\left( \right)=\alpha_{2}+\alpha_{2\to3}+\alpha_{3\to4}+\ldots+\alpha_{8\to9}+g\left( X \right)$$

where *X* represents predictors and α are baseline probabilities. For sweetness, the predictors were as follows:

$$g_{S}\left( X \right)=\frac{Sugar\cdot S_{max-Sugar}}{Sugar+{SSugar}_{50}}+{SL}_{S-Fat}\cdot Fat$$

For creaminess, the predictors were as follows:

$$g_{C}\left( X \right)={SL}_{C-Fat}\cdot Fat+{SL}_{C-Sugar}\cdot Sugar$$

***The differential odds model***

The difference between the proportional and the differential odds models lays in the function of the predictor, *g(X)* which for the proportional odds model is the same for all categories, but is allowed to vary for the differential odds model (see Eq. 4).

$$f_{2}\left( \right)=\alpha_{2}+g_{2}\left( X \right)$$

$$f_{3}\left( \right)=\alpha_{2}+\alpha_{2\to3}+g_{3}\left( X \right)$$

$f_{4}\left( \right)=\alpha_{2}+\alpha_{2\to3}+\alpha_{3\to4}+g_{4}\left( X \right)$ (4)

*…*

$$f_{9}\left( \right)=\alpha_{2}+\alpha_{2\to3}+\alpha_{3\to4}+\ldots+\alpha_{8\to9}+g_{9}\left( X \right)$$

For pleasantness, the predictors were as follows:

$$g_{P2}\left( X \right)=\frac{Sugar\cdot P_{max-Sugar}}{Sugar+{PSugar}_{50}}+\frac{Fat\cdot P_{max-Fat}}{Fat+{PFat}_{50}}+{IP}_{Sugar-Fat}\cdot\left( {wIP}_{Sugar-Fat}\frac{Sugar}{Max(Sugar)}+\frac{Fat}{Max(Fat)} \right)$$

$$g_{P3}\left( X \right)=\frac{Sugar\cdot P_{max-Sugar}}{Sugar+{PSugar}_{50}}+\frac{Fat\cdot P_{max-Fat}\cdot\beta_{C3}}{Fat+{PFat}_{50}}+{IP}_{Sugar-Fat}\cdot\left( {wIP}_{Sugar-Fat}\frac{Sugar}{Max(Sugar)}+\frac{Fat}{Max(Fat)} \right)$$

$$g_{P4}\left( X \right)=\frac{Sugar\cdot P_{max-Sugar}}{Sugar+{PSugar}_{50}}+\frac{Fat\cdot P_{max-Fat}\cdot\beta_{C3}\cdot\beta_{C4}}{Fat+{PFat}_{50}}+{IP}_{Sugar-Fat}\cdot\left( {wIP}_{Sugar-Fat}\frac{Sugar}{Max(Sugar)}+\frac{Fat}{Max(Fat)} \right)$$

$$g_{P5/P6}\left( X \right)=\frac{Sugar\cdot P_{max-Sugar}\cdot\beta_{Sugar5}}{Sugar+{PSugar}_{50}}+\frac{Fat\cdot P_{max-Fat}\cdot\beta_{C3}\cdot\beta_{C4}}{Fat+{PFat}_{50}}+{IP}_{Sugar-Fat}\cdot\left( {wIP}_{Sugar-Fat}\frac{Sugar}{Max(Sugar)}+\frac{Fat}{Max(Fat)} \right)$$

$$g_{P7/P8/P9}\left( X \right)=\frac{Sugar\cdot P_{max-Sugar}\cdot\beta_{Sugar5}}{Sugar+{PSugar}_{50}}+\frac{Fat\cdot P_{max-Fat}\cdot\beta_{Fat3}\cdot\beta_{Fat4}\cdot\beta_{Fat7}}{Fat+{PFat}_{50}}+{IP}_{Sugar-Fat}\cdot\left( {wIP}_{Sugar-Fat}\frac{Sugar}{Max(Sugar)}+\frac{Fat}{Max(Fat)} \right)$$

***Probability predictions***

Independent of if the model is implemented in terms of the proportional or differential odds model, the probability of a score is expressed as:

$$Pr\left( y_{in}=1 | \eta_{i} \right)=1-Pr\left( y_{in}\geq2 | \eta_{i} \right)$$

$$Pr\left( y_{in}=2 | \eta_{i} \right)=Pr\left( y_{in}\geq2 | \eta_{i} \right)-Pr\left( y_{in}\geq3 | \eta_{i} \right)$$

$$Pr\left( y_{in}=3 | \eta_{i} \right)=Pr\left( y_{in}\geq3 | \eta_{i} \right)-Pr\left( y_{in}\geq4 | \eta_{i} \right)$$

$$Pr\left( y_{in}=4 | \eta_{i} \right)=Pr\left( y_{in}\geq4 | \eta_{i} \right)-Pr\left( y_{in}\geq5 | \eta_{i} \right)$$

$$Pr\left( y_{in}=5 | \eta_{i} \right)=Pr\left( y_{in}\geq5 | \eta_{i} \right)-Pr\left( y_{in}\geq6 | \eta_{i} \right)$$

$$Pr\left( y_{in}=6 | \eta_{i} \right)=Pr\left( y_{in}\geq6 | \eta_{i} \right)-Pr\left( y_{in}\geq7 | \eta_{i} \right)$$

$$Pr\left( y_{in}=7 | \eta_{i} \right)=Pr\left( y_{in}\geq7 | \eta_{i} \right)-Pr\left( y_{in}\geq8 | \eta_{i} \right)$$

$$Pr\left( y_{in}=8 | \eta_{i} \right)=Pr\left( y_{in}\geq8 | \eta_{i} \right)-Pr\left( y_{in}\geq9 | \eta_{i} \right)$$

$Pr\left( y_{in}=9 | \eta_{i} \right)=Pr\left( y_{in}\geq9 | \eta_{i} \right)$

**S2. Parameter estimates with relative standard errors (RSE) for the proportional odds models for sweetness and creaminess score and the differential odds model for the pleasantness score.**

|  | Parameter Description | Parameter | Estimate | RSE |
| --- | --- | --- | --- | --- |
| Sweetness Score | Logit of score >1 | *α_S1_* | -0.764 | 70% |
|  | Logit of score =2 | *α_S2->3_* | -1.12 | 10% |
|  | Logit of score =3 | *α_S3->4_* | -1.00 | 13% |
|  | Logit of score =4 | *α_S4->5_* | -0.829 | 9.1% |
|  | Logit of score =5 | *α_S5->6_* | -0.740 | 9.9% |
|  | Logit of score =6 | *α_S6->7_* | -0.797 | 10.7% |
|  | Logit of score =7 | *α_S7->8_* | -0.979 | 8.0% |
|  | Logit of score =8 | *α_S8->9_* | -1.23 | 6.6% |
|  | Maximal effect of sugar | *S_max-Sugar_* | 8.32 | 4.9% |
|  | Sugar giving half of S_max,Sugar_ | *SSugar_50_* | 7.89 | 14% |
|  | Slope of fat effect | *SL_S-Fat_* | 0.004 | 140% |
| Creaminess Score | Logit of score >1 | *α_C1_* | 1.06 | 62% |
|  | Logit of score =2 | *α_C2->3_* | -1.46 | 6.0% |
|  | Logit of score =3 | *α_C3->4_* | -1.06 | 8.9% |
|  | Logit of score =4 | *α_C4->5_* | -0.83 | 11% |
|  | Logit of score =5 | *α_C5->6_* | -0.846 | 13% |
|  | Logit of score =6 | *α_C6->7_* | -0.902 | 14% |
|  | Logit of score =7 | *α_C7->8_* | -1.12 | 15% |
|  | Logit of score =8 | *α_C8->9_* | -1.43 | 11% |
|  | Slope of fat effect | *SL_C-Fat_* | 0.186 | 14% |
|  | Slope of sugar effect | *SL_C-Sugar_* | 0.049 | 16% |
| Pleasantness Score | Logit of score >1 | *α_P1_* | -0.482 | 165% |
|  | Logit of score =2 | *α_P2->3_* | -1.36 | 11% |
|  | Logit of score =3 | *α_P3->4_* | -1.2 | 14% |
|  | Logit of score =4 | *α_P4->5_* | -0.865 | 6.6% |
|  | Logit of score =5 | *α_P5->6_* | -0.627 | 35% |
|  | Logit of score =6 | *α_P6->7_* | -0.661 | 13% |
|  | Logit of score =7 | *α_P7->8_* | -1.21 | 19% |
|  | Logit of score =8 | *α_P8->9_* | -1.34 | 12% |
|  | Maximal effect of sugar | *P_max-Sugar_* | 7.96 | 105% |
|  | Sugar giving half of P_max-Sugar_ | *PSugar_50_* | 9.86 | 120% |
|  | Maximal effect of fat | *P_max-Fat_* | 2.41 | 61% |
|  | Fat giving half P_max-Fat_ | *PFat_50_* | 8.71 | 72% |
|  | Interaction of sugar and fat | *IP_Sugar-Fat_* | -2.31 | 44% |
|  | Weight of sugar versus fat on IP_Sugar-Fat_ | *wIP_Sugar-Fat_* | 1.06 | 160% |
|  | Differential effect ≥5, Sugar | *β_Sugar5_* | 0.928 | 7.7% |
|  | Differential effect ≥3, Fat | *β_Fat3_* | 1.23 | 12% |
|  | Differential effect ≥4, Fat | *β_Fat4_* | 1.26 | 14% |
|  | Differential effect ≥7, Fat | *β_Fat7_* | 1.19 | 11% |
| Variance | Sweetness baseline* | *ω^2^_S_* | 1.34 | 35% |
|  | Creaminess baseline* | *ω^2^_C_* | 4.21 | 31% |
|  | Pleasantness baseline* | *ω^2^_P_* | 4.21 | 28% |
|  | S_max-Sugar_ | *ω^2^_Smax-Sugar_* | 0.207 | 33% |

* Variance is implemented as additive on logit scale

A B C D

**Figure S1: Distribution of Maximum Sweetness Score with Highest Level of Sugar (20%) with 0% fat (A), 4% fat (B), 11% fat (C), and 37.5% fat (D)**

A B C D

**Figure S2: Distribution of Maximum Creaminess Score with Highest Level of Fat (37.5%) with 0% sugar (A), 5% sugar (B), 10% sugar (C), and 20% sugar (D)**

A B C D

**Figure S3: Distribution of Maximum Pleasantness Score with Highest Level of Fat (37.5%) with 0% sugar (A), 5% sugar (B), 10% sugar (C), and 20% sugar (D)**

A B C D

**Figure S4: Distribution of Maximum Pleasantness Score with Highest Level of Sugar (20%) with 0% fat (A), 4% fat (B), 11% fat (C), and 37.5% fat (D)**
